# Impacts of the COVID-19 pandemic on private hospitals in Brazil

**DOI:** 10.31744/einstein_journal/2023AO0174

**Published:** 2023-10-10

**Authors:** Andrizio Alexandrino de Morais, Ana Maria Malik, Gonzalo Vecina

**Affiliations:** 1 Fundação Getulio Vargas Escola de Administração de Empresas de São Paulo São Paulo SP Brazil Fundação Getulio Vargas , Escola de Administração de Empresas de São Paulo , São Paulo , SP , Brazil .

**Keywords:** Pandemics, COVID-19, Coronavirus infections, Hospitals, private, Cost of illness, Health care costs, Brazilian Health Surveillance Agency, Brazil

## Abstract

**Objective:**

To analyze the operational and/or financial impacts of regulatory measures implemented by the National Agency for Supplementary Health and government health departments in response to the COVID-19 pandemic on a group of 118 private hospitals affiliated with the National Association of Private Hospitals.

**Methods:**

This study used a quantitative methodological design of descriptive, cross-sectional, and retrospective studies, utilizing secondary data provided by the National Agency for Supplementary Health.

**Results:**

There was a -20.1% reduction in hospital admissions and hospital occupancy rate and a decrease of -4.4 percentage points in earnings before interest, taxes, depreciation, and amortization margins. Additionally, the average length of hospital stay increased by +0.5, while total expenses for hospital leaves and total net revenue for hospital leaves increased by +39.4% and +23.6%, respectively. Comparing the participation of the different International Classification of Diseases, according to ICD-10 in 2019 and 2020, revealed the following variations in percentage points of hospitalizations. Hospitalizations for infectious diseases (including COVID-19), treatment of neoplasms, and pregnancy increased by +2.1, +2.4, and +1.2, respectively. However, hospitalizations for respiratory diseases decreased by -4.1.

**Conclusion:**

The most critical period of the pandemic required the redirection of activities to concentrate efforts on caring for COVID-19 cases. This situation highlighted the non-prioritization of primary care, as many problems presented by patients not affected by COVID-19 had to be referred to emergency services when and if appropriate.

## In Brief

The COVID-19 pandemic has significantly impacted hospital performance. To assess its effects on hospital indicators, we studied a sample of over 100 facilities. These facilities are members of a private hospital association, considered quality institutions with higher-tier socioeconomic patients, and are mostly financed by private insurers. We utilized publicly available data for 2020, the year when the pandemic effect was most acute

## Highlights

Sanitary restriction measures had a strong impact on usual performance indicators from a selected group of private hospitals in Brazil.

P۪۪regnancies and deliveries continued to constitute an important proportion of hospital admissions because they did not depend on external restrictions.

H۪۪ospital costs increased due to increased utilization of more expensive Personal Protection Equipment (due to lack of availability or much higher demand).

Article extracted from the master’s dissertation presented to the Master´s Program in Management for Competitiveness at *Fundação Getulio Vargas* , São Paulo, SP, in 2022.

## Visual Abstract

**Figure:**
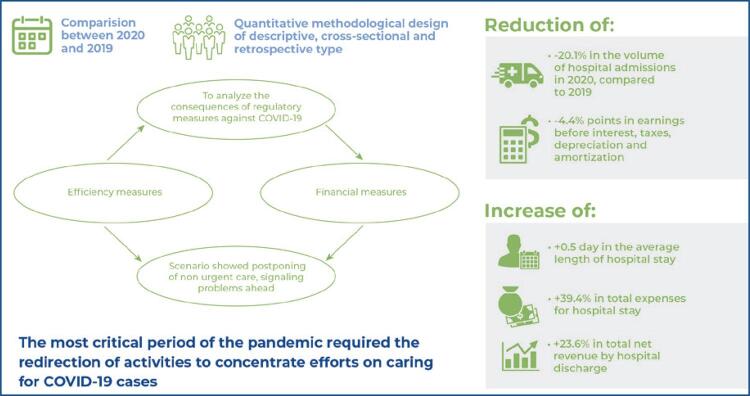

## INTRODUCTION

The first case of coronavirus disease 2019 (COVID-19) in Brazil was reported in February 2020 in a Brazilian patient from the State of São Paulo who was returning from Italy. ^( 1 )^ According to the press, he was treated at the emergency room of a reference hospital in the state capital and sent back to his residence. The declaration of the pandemic as a Public Health Emergency of National Interest (PHENI - https://bvsms.saude.gov.br/bvs/saudelegis/gm/2020/prt0188_ 04_02_2020.html) triggered a series of public policies in Brazil and worldwide, based on the relatively limited knowledge available at that time. For example, masks were considered as inputs for hospital use only, and the demand for health services, including diagnostic centers, was discouraged for any cause that did not appear to be the new disease ( *i.e* ., respiratory symptoms). Additionally, primary care services in the primary health and outpatient network/medical offices remained closed, except for urgent/emergency cases.

The research question that motivated this article was as follows: What happened to the performance of private hospitals in 2020, the first year of the COVID-19 pandemic? Performance was defined in terms of production indicators and financial performance. Due to the availability of organized data and to the peculiar characteristics of this universe, the authors decided to conduct a more detailed study of the 118 hospitals associated with the National Association of Private Hospitals (ANAHP - *Associação Nacional de Hospitais Privados* ). These hospitals respond for almost 4% of private hospitals in Brazil and approximately 10% of the country’s private beds, and data from 2020 were compared to those from 2019. However, since ANAHP hospitals do not represent the entire spectrum of private healthcare facilities in the country, the discussion also incorporates data from other private hospitals, specifically those owned by private health insurance companies that operate under a verticalized business model.

In Brazil, there were approximately 6.300 hospitals and less than 450.000 beds in 2020, which were unevenly distributed throughout the territory. ^( 2 )^ Data from the Brazilian Federation of Hospitals (BFH) indicate that the number of beds decreased between 2010 and 2020. Of these hospitals, 3.600 (more than 50%) were private. All of these data create a peculiar situation for private facilities, both for those primarily financed by the public sector of the Unified Health System (SUS - *Sistema Único de Saúde* ) and those that heavily rely on health insurance plans and/or can also be paid directly by patients in an out-of-pocket model.

## OBJECTIVE

To analyze whether the regulatory measures of the National Agency for Supplementary Health and Government Health departments in response to the COVID-19 pandemic have had operational and/or financial impacts on a group of 118 private hospitals affiliated with the National Association of Private Hospitals.

## METHODS

The ANAHP was established in 2001 to “defend the interests of this sector… expand the improvements achieved by private institutions beyond the borders of Supplementary Health, thus favoring all Brazilians… it plays a strategic role in the scenario…the unfolding of themes essential to the sustainability of the system”. ^( 3 , 4 )^ In addition to explicit advocacy in its mission, this association has established specific requirements to become an associate. These requirements include being a private hospital, for profit or non profit, not being owned or managed by Private Payor organizations, having at least 60% of its gross revenue derived from private paying sources, and being accredited from either an international organization or the National Accreditation Organization at level III. ^( 4 )^

To answer the research question, a possible strategy was to use data publicly available on the ANAHP website, as there is an agreement related to the confidentiality of data between hospitals and the association. This article adopts a quantitative, descriptive, cross-sectional, and retrospective research design, utilizing secondary data provided by the ANAHP. ^( 5 - 12 )^

This study analyzed the results of the variation in different indicators by comparing the years 2020 and 2019. The compared indicators were as follows: production indicators, hospital occupancy rate (%), ^( 13 )^ average length of stay–hospital length of stay (days), ^( 13 )^ epidemiological profile of hospital admissions according to the International Classification of Diseases (ICD10); ^( 13 , 14 )^ financial indicators, such as earnings before interest, taxes, depreciation, and amortization (EBITDA) margin, ^( 15 )^ net revenue, and total expenditure per patient-day. ^( 5 - 12 )^

The survey considered the indicators between January 1 and December 31, 2020, and compared them with those from January 1 to December 31, 2019.

Two limitations are recognized *a priori*: 1) these are secondary data from hospitals, sent voluntarily to ANAHP, without any prior accuracy certification; and 2) among the 2020 data are those corresponding to the first 9 months of the pandemic (March to December), before the availability of vaccines, which started in 2021, considerably changing the scenario of sanitary conditions. Despite these restrictions, we consider the data analyzed here valid for understanding the period under study.

## RESULTS

Data from the Association’s hospitals showed a negative variation of 20.1% in the volume of hospital admissions in 2020 compared to the same sample of hospitals in 2019. Comparing the participation of the different International Classification of Diseases (ICD), according to chapter ICD-10 in 2019 and 2020, the following notable variations were observed. There was an increase of 2.1 percentage points in the proportion of hospitalizations related to infectious diseases (which includes COVID-19). Similarly, the proportion of hospitalizations for the treatment of neoplasms increased by 2.4 percentage points. Conversely, there was a reduction of 4.1 percentage points in the proportion of hospitalizations compared to the previous year; however, the advanced life support (ALS) in hospitals increased from 6.1 days in 2019 to 9 days in 2020. Further, there was an increase of 1.2 percentage points in the proportion of hospitalizations for pregnancy in 2020 compared to 2019.

These profile changes are directly related to the temporary suspension of surgical procedures, sanitary measures of the authorities with decrees recommending the suspension or the deferment of ellective surgeries, and the fear of contamination by health professionals themselves and by beneficiaries of health insurance plans. Although not within the scope of this study, the oscillation of the mortality rate in this sample was almost nonexistent. ^( 5 )^

Public data show the resilience of the Brazilian health system as a whole with postponements of procedures and the reduction in the use of services during this period. ^( 16 )^ The reality evidenced in this article does not reflect these data, as it does not consider the Brazilian population as a whole, but only this specific sample. ^( 16 )^

### Specific indicators

#### Occupancy percentage

**Figure 1:**
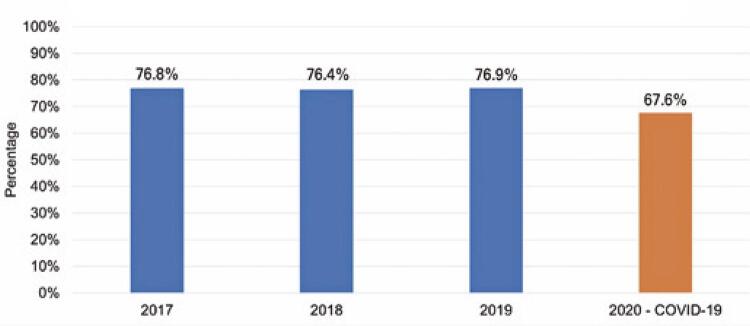
Comparative distribution of the historical series of the hospital occupancy rate of hospitals affiliated to ANAHP

Source: elaborated and adapted by the authors based on the ANAHP data. ^( 11 )^

As shown in figure 1 , from 2017 to 2019, the occupancy rate of hospitals affiliated with the ANAHP ranged from 76.4% in 2018 to 76.9% in 2019. In 2020, considering the first 3 months of the year (pre-pandemic), this indicator was at 70.2%. In the initial phase of the pandemic, characterized by uncertainty and fear among patients and healthcare professionals, particularly in the second quarter of 2020, the occupancy rate declined (including hospitalizations for COVID-19) to 59.4%, recovering to 72.4% in the last quarter of the year. Hospital occupancy rates can interfere with the cash flows of private hospitals. ^( 17 )^

ANAHP: Associação Nacional de Hospitais Privados.

**Figure 2:**
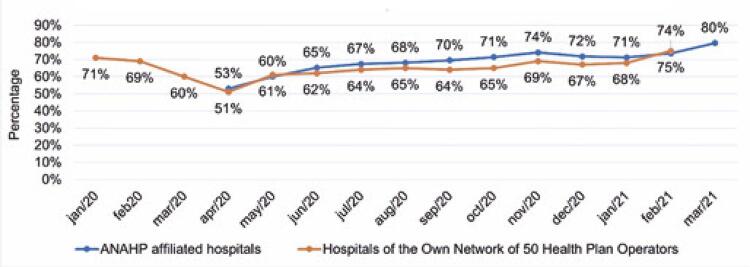
Distribution of the historical series of the general hospital occupancy rate among hospitals affiliated with ANAHP and hospitals of 50 HPS with their own hospital network

Source: Elaborated and adapted by the authors based on ANAHP and NPS data. ^( 5 - 12 , 18 )^

ANAHP: Associação Nacional de Hospitais Privados; HPS: Health Plan Operators.

Figure 2 shows the result of comparing the occupancy rate of hospitals affiliated with ANAHP to that of a group of hospitals belonging to 50 large Health Plan Operators (HPS). ^( 5 - 12 , 18 )^

In both groups, there was a large reduction in hospital occupancy in April 2020, with gradual growth in the subsequent months. However, in the historical series, the occupancy rate was generally higher in private hospitals affiliated with the ANAHP.

## Average length of stay

**Figure 3:**
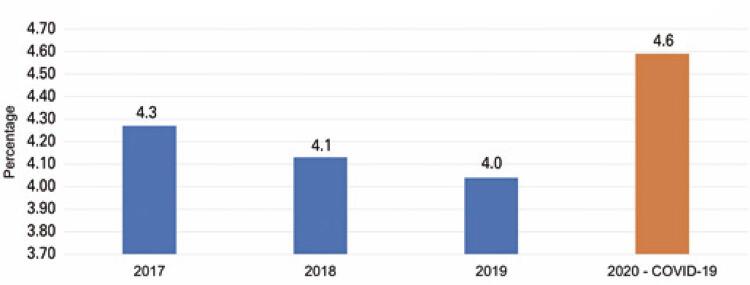
Distribution of the historical series of the average length of stay in hospitals affiliated with ANAHP

Source: elaborated and adapted by the authors based on the ANAHP data. ^( 11 )^

Hospital efficiency studies show that in maintaining case-mix conditions, the lowest average length of stay corresponds to the most efficient management. ^( 19 )^ In fact, among hospitals affiliated with the ANAHP, between 2017 and 2019, the average length of stay fell annually from 4.3 to 4 days. However, with the changes (including those related to the type of cases treated) that occurred in 2020, the overall average length of stay increased to 4.6 days, as depicted in figure 3 . A higher average length of stay leads to increased resource consumption and reduced productivity. ^( 20 )^

ANAHP: Associação Nacional de Hospitais Privados.

However, the relevance of intensive care unit (ICU) beds during the study period should be considered. ^( 21 )^Table 1 shows the variation in the average length of stay in these beds of hospitals affiliated with ANAHP .

**Table 1:** Average hospital stay, in days, in intensive care, semi-intensive care, pediatric intensive care unit, and neonatal intensive care unit in the four quarters

ICU	2019				2020			

	1st Quarter	2nd Quarter	3rd Quarter	4th Quarter	1st Quarter	2nd Quarter	3rd Quarter	4th Quarter
Adult ICU	5.9	5.5	5.7	5	5	5.7	5.5	5.4
Semi-intensive ICU	5.9	6	5.9	5.6	5.6	5.7	5.2	5.5
Pediatric ICU	6.9	6,6	5.7	5.9	6.4	8	6.1	6.1
Neonatal ICU	14	12.9	14.4	14	14.1	15.9	13.4	13.1

Source: Prepared by the authors based on data from ANAHP Technical Notes (2021). ^( 11 )^

ICU: intensive care unit.

In general, there was minimal variation in the mean length of stay in ICU beds, except for pediatric beds.

## Revenue and expense indicators

Discussing revenue and expenditure in the context of the first year of the pandemic requires consideration of the changes in available knowledge. For example, in the initial months, when the widespread belief was that the contagion was due to contact, there was a global rush to purchase personal protective equipment (PPE) that led to increased prices due to their low availability in the national market and the law of supply and demand. A survey of the impact of the consumption of PPE due to COVID-19 conducted by the association with its affiliates revealed that prices for the PPE items described in the table below experienced an average increase of 309%, with a consequent impact on hospital costs ( Table 2 ). ^( 22 )^

**Table 2:** Differences in the use of personal protective equipment/day during the period from 2019 to 2020, comparing care for patients with COVID-19 and those provided to other patients

Disposable material	Amount of PPE needed to treat one patient diagnosed with COVID-19 for 1 day		Amount of PPE needed to treat one patient with other diagnoses for 1 day		Change in unitary cost of PPE April/2019 *versus* April/2020 %

	ICU	Inpatient Units	ICU	Inpatient Units	
N95 Masks	8	3	2	1	405
Surgical masks	24	25	19	20	562
Cap	21	17	3	2	63
Shoe covers	14	5	1	1	337
Apron	33	16	4	2	178
Total PPE per day	100	66	28	26	

Source: elaborated and adapted by the authors based on the ANAHP data. ^( 22 )^

PPE: personal protective equipment; ICU: intensive care unit.

**Figure 4:**
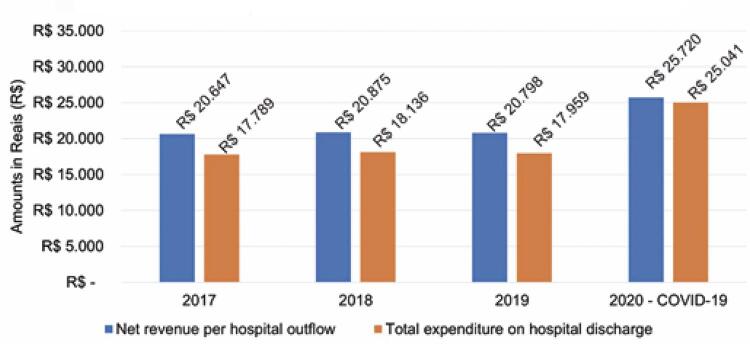
Distribution of the historical series of net revenue and total expenditure per hospital discharge

Source: elaborated and adapted by the authors based on the ANAHP data. ^( 5 )^

Based on data from ANAHP, figure 4 shows a comparison of the revenue per hospital discharge *versus* the total expenditure by hospital leave in 2020 *versus* 2019; ^( 5 )^ there was a variation of 23.6% and 39.4%, respectively.

**Figure 5:**
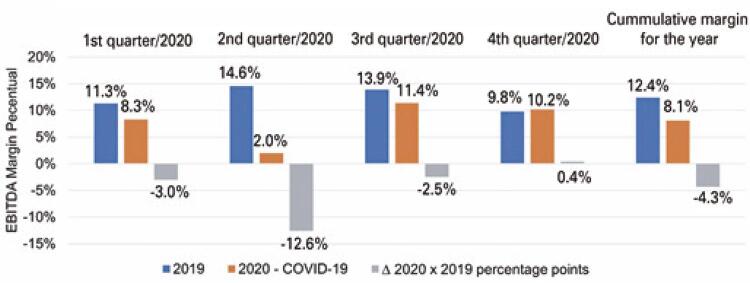
Distribution of the comparative Historical series of quarterly and annual EBITDA margins of hospitals affiliated with ANAHP ANAHP: Associação Nacional de Hospitais Privados.

Source: elaborated and adapted by the authors based on ANAHP data. ^( 5 )^

The EBITDA margin demonstrates a company’s ability to generate operating cash flow. In this case, the reduction shown in figure 5 indicates a worsening of this capacity and a consequent decrease in the financial results of hospitals.

Because the ANAHP does not make EBITDA available in the local currency (R$), it was not possible to report the financial impact from a monetary perspective. However, it can be estimated that due to the reduction in the number of elective procedures, hospitals have faced a decrease in revenue, concomitant with the maintenance of their fixed costs.

## DISCUSSION

In the context of a public health emergency, discussions regarding testing commenced, ranging from rapid tests to reverse-transcriptase polymerase chain reaction (RT-PCR) tests, enabling drive-through mechanisms. Additionally, the National Agency for Supplementary Health introduced regulations pertaining to the care rendered to beneficiaries of health plans in the face of the pandemic. Initially, a technical note ^( 23 )^ was issued suspending the performance of elective procedures between March and June 2020. Subsequently, a Normative Resolution (NR) ^( 24 )^ was published, mandating coverage for health plan tests to assist in diagnosis. Finally, another NR was published, updating the list of procedures and events that require minimum and mandatory coverage for the assistance of health plan beneficiaries. ^( 25 )^

In 2020, the National Health Surveillance Agency (ANVISA - *Agência Nacional de Vigilância Sanitária* ) issued regulations on the import of supplies that were lacking in the country. ^( 26 , 27 )^ From the end of the same year, it also payed its ordinary role in approving vaccines.

Additionally, there was a significant mobilization and implementation of appropriate health policies in response to the public health emergency, such as quarantine, curfews, opening field hospitals, isolation/social distancing, and an emergency program to maintain employment and income at different levels of government. Moreover, collaboration between the public and private sectors was observed at the national level, with variations among states and municipalities. The opening of field hospitals was the main early public policy to improve the supply of beds for access to healthcare during the COVID-19 pandemic in Brazil. ^( 27 )^ On the other hand, some states and municipalities favored the requisition of private beds.

The data collected from both the ANAHP and verticalized hospitals did not indicate that there was excessive use of beds, as seen in beds financed directly or indirectly by the public sector. Overall, the occupancy rate was not under national pressure. In the states where demand was higher, as in the northern region in general (particularly in Manaus), the healthcare network was under pressure. However, because data from the ANAHP reports do not support this assumption, further studies are required.

Before the onset of the health crisis, it was common knowledge that the supply of beds, particularly in the ICUs, was higher in the private sector than in the public sector. ^( 27 )^ Data recorded on the website of the Ministry of Health in early 2020 indicated a ratio of 32.8 ICU beds per 100,000 beneficiaries in the private network and seven beds per 100,000 inhabitants in the public network. This disproportion led some states (Espírito Santo, Tocantins) and municipalities (Curitiba) to intervene in the private network and purchase additional capacity in the private network (São Paulo). During the pandemic, these issues were addressed throughout the country with varying degrees of success.

However, the ANAHP data showed no significant impacts on the private hospital network in terms of bed occupancy or average length of stay. The low interference due to the pandemic in the average length of stay in these hospitals was surprising because an increase in the average length of stay was expected in the information disclosed. Overall, the patients diagnosed with COVID-19 had longer stays. Perhaps because they reserved beds for other procedures, the data did not indicate this expected effect by referring to the management of patients with COVID-19 for different services.

On the other hand, financial data showed the simultaneous impact of revenue loss and increased expenditure. Expenditures grew not only because of increased consumption of PPE but also due to the price increase practiced by different suppliers during the initial period of the pandemic. There was also an increase in the use of medications in the intubation processes of patients and an increase in uncontrolled prices in the hospital market.

## CONCLUSION

The pandemic has provided valuable lessons because coping with the most critical period required the redirection of activities aiming to concentrating efforts on the care of cases resulting from the pandemic. Many issues presented by patients not affected by COVID-19 had to be addressed at the doors of emergency services, as appropriate, highlighting the non-prioritization of primary care. In the public network, which was not the focus of this study, there are data that show that the consequences of epidemiological and production profiles were more severe. However, in private networks, the impact of defunding generated by a fall in revenue and increased expenditure calls for further studies, using primary data.

The data collected and analyzed from this association showed that this group of private hospitals experienced a decline in surgical productivity, decreased revenues, increased fixed costs, and significantly reduced profit margins in the first year of the COVID-19 pandemic.
